# Development of a coagglutination kit as a rapid test for diagnosing Newcastle disease in poultry

**DOI:** 10.14202/vetworld.2020.1719-1724

**Published:** 2020-08-28

**Authors:** Muhammad Kholish Naf’an, Kurniasih Kurniasih, Tri Untari, Yos Adi Prakoso

**Affiliations:** 1 Student of Master of Sciences Degree, Faculty of Veterinary Medicine, University of Gadjah Mada, Yogyakarta, Indonesia; 2 Department of Pathology, Faculty of Veterinary Medicine, University of Gadjah Mada, Yogyakarta, Indonesia; 3 Department of Microbiology, Faculty of Veterinary Medicine, University of Gadjah Mada, Yogyakarta, Indonesia; 4 Faculty of Veterinary Medicine, University of Wijaya Kusuma Surabaya, East Java, Indonesia

**Keywords:** coagglutination kit, histopatology, Newcastle disease, rapid test, reverse transcription-polymerase chain reaction

## Abstract

**Background and Aim:**

Newcastle disease (ND) is a viral infection that causes high mortality and economic loss in the poultry industry. The Office International des Epizooties (OIE) recommends several diagnostic methods for the detection of ND, including isolation and molecular tests. However, these detection methods are time-consuming and highly expensive. Therefore, this study was conducted to develop a coagglutination kit as a novel diagnostic tool for ND in the poultry industry.

**Materials and Methods:**

Two adult male New Zealand White rabbits weighing 2.5 kg were vaccinated using ND life vaccine intraperitoneally. The vaccination was conducted once a week for 4 weeks with multilevel doses. Rabbits’ serum was collected at week 6 and inactivated at 56°C for 30 min. The serum was precipitated using ammonium sulfate and reacted with protein A of Staphylococcus aureus to produce the agglutination kit for detecting ND virus. A total of 25 chickens suspected with ND infection from a local poultry farm in Yogyakarta were used as the test samples. The chickens were necropsied, and the brain, spleen, lung, intestine, and feces were collected. Half of these organs were subjected to tests using the coagglutination kit and reverse transcription-polymerase chain reaction (RT-PCR). The other half was processed for histopathology. Data were analyzed qualitatively.

**Results:**

Of the 25 samples, 13 (52%) were positive for ND infection when tested using both the ND coagglutination kit and RT-PCR. The positive samples also exhibited several histopathological changes, including perivascular cuffing surrounding the cerebral blood–brain barrier, hemorrhagic pneumonia, splenitis, and necrotic hemorrhage enteritis.

**Conclusion:**

This study confirmed that the ND coagglutination kit could be used as a novel diagnostic tool for the detection of ND virus infection in the poultry industry.

## Introduction

Newcastle disease (ND) is an infectious poultry disease caused by a virus belonging to the *Paramyxoviridae* family, serogroup *Avian paramyxovirus* type 1 (APMV-1). This disease was first reported in Java, Indonesia, in 1926, when it caused high mortality, morbidity, and economic losses in the local poultry industry [1]. In the United States, it has been reported that the impact of ND infection in the poultry industry resulted in the culling of a total of 3.16 million birds or an estimated economic loss reaching $121 million [2]. Vaccination and good management practice are the standard procedures to prevent ND infection within the flocks [3].

ND commonly causes several clinical signs, including respiratory symptoms and neurologic and digestive problems. Moreover, ND can manifest clinical signs similar to those of avian influenza (AI) [4]. As a result, the Office International des Epizooties (OIE) has recommended several standard diagnostic methods for the detection of ND, including virus isolation and molecular tests [5]. However, both these detection methods are time-consuming and expensive and require reliable operators.

Today, the poultry industry requires rapid diagnostic tools with high specificity and sensitivity. In this regard, a coagglutination test can be considered as one of the diagnostic tools for the detection of human and animal diseases. The diagnostic principle of a coagglutination test involves the serological mechanism. This method is simple, rapid, and inexpensive compared with other ND detection methods. Although coagglutination has been used to diagnose microsporidiosis in humans [6], it has never been applied to diagnose ND virus infection in poultry.

Therefore, this study was conducted to develop a coagglutination kit as a novel diagnostic tool for detecting ND in the poultry industry.

## Materials and Methods

### Ethical approval

All the animal procedure was approved and monitored by Ethical Clearance Committee of Faculty of Veterinary Medicine, University of Gadjah Mada, Yogyakarta, Indonesia (Approval no. 0005/EC-FKH/Int./2020).

### Time and place of study

The study was conducted from January 2019 to March 2020. The animal models were maintained in the Department of Pathology, Faculty of Veterinary Medicine, University of Gadjah Mada, Yogyakarta, Indonesia. The antibody purification and molecular test were performed in the Laboratory of Microbiology and Virology, Station of Fish Quarantine and Quality Control, Yogyakarta, Indonesia.

### Immunoglobulin production and purification

Two adult male New Zealand White rabbits weighing 2.5 kg were vaccinated using the ND life vaccine (Medivac ND Lasota, Medion) intraperitoneally. The vaccination was conducted once a week for 4 weeks with multilevel doses (0.5, 1.00, 2.00, and 3.00 mL). In the 6^th^ week, 10 mL of rabbit blood was collected using a 24 G disposable syringe through an auricular vein.

The production and purification of polyclonal antibody from rabbit IgG were performed according to a previously described method [7] with minor modifications. The collected blood was incubated at 37°C in an incubator until the separation of serum. The serum was collected, inactivated at 56°C, and then stored in the refrigerator. Serum purification was done using ammonium sulfate. The mixture was then centrifuged at 6000 rpm for 30 min, and the supernatant was discarded. The resulting precipitate was then washed 3 times using NaCl. Next, the precipitate was dialyzed using 20 mL of molecular weight cutoff (6-8 kDa) (Merckmillipore, catalog number 71746), soaked in phosphate-buffered saline (PBS; pH 7.2) for 12 h, and this process was repeated 3 times. The final product obtained from this process is a pure immunoglobulin solution.

### Isolation of protein A of Staphylococcus aureus and immunoglobulin binding

*S. aureus* isolate (ATCC 12598) was cultured on Tryptic Soy Agar (TSA) and incubated at 37°C for 24 h. The isolate was then collected and mixed with PBS (pH 7.2). The collected isolate was washed 3 times using PBS, followed by the addition of 0.5% formalin and incubation overnight at room temperature (25°C). The resulting pellet was heated using a water bath at 80°C for 15 min and then cooled rapidly. The pellet was mixed with PBS and made into a suspension at 10% concentration (w/v) [8]. Next, the purified rabbit immunoglobulin was added to the pellet and the mixture was incubated overnight at room temperature. The obtained cocktail suspension was then centrifuged at 3000 rpm for 10 min. The supernatant was discarded, and the resulting precipitate was mixed with PBS (pH 7.2). Finally, the coagglutination kit was ready to use [9].

### Sample preparation and coagglutination test procedure

A total of 25 chickens suspected with ND infection were collected from a local poultry farm in Yogyakarta. The chickens were necropsied, and the brain, spleen, lung, intestine, and feces were collected. These specimens were divided into two different portions. The first portion was stored in a sterile plastic bag for the coagglutination and molecular tests, and the second portion was stored in 10% neutral buffered formalin (NBF) for histopathology.

For the coagglutination test, the organs were separately blended and mixed with PBS (1:1). The mixture of organ and PBS was centrifuged at 8000 rpm for 10 min, and the supernatant was used as the test specimen. Next, 50 μL of the supernatant was dropped on the surface of a glass slide and reacted with 50 μL of the coagglutination kit. The reaction was observed for 5-10 min. A sandy agglutination indicated a positive reaction, whereas the absence of agglutination indicated a negative reaction.

### Histopathology

Fixed organs were processed routinely for histopathology using hematoxylin and eosin (H&E) staining. H&E staining was conducted following the procedure described in a previous study [10].

### Reverse transcription-polymerase chain reaction (RT-PCR)

For comparison, the test specimens were also subjected to RT-PCR. The samples were tested using the primer obtained from the Disease Investigation Center, Lampung, F: 5′-GCTGTATCTGTCTGACAAGCTCTC-3′ and R: 5′-AGGTTGAGTTCTACACCAACCTGT-3′. RNA extraction was performed using the High Pure Viral Nucleic Acid Kit for Rapidly Isolate Highly Purified Viral DNA and RNA from Roche. For amplification, the GoTaq^®^ Green Master Mix and Nuclease-Free Water from PROMEGA were used. The mixture was inserted into the thermocycler to synthesize cDNA at 50°C for 30 min (1 cycle), followed by predenaturation at 94°C for 5 min (1 cycle). Next, denaturation was performed at 94°C for 40 s, followed by annealing at 55°C for 30 s, and elongation at 72°C for 60 s (35 cycles). Finally, postelongation was performed at 72°C for 4 min (1 cycle) [11].

### Statistical analysis

The results of coagglutination reaction, histopathology, and RT-PCR were analyzed using simple descriptive qualitative methods. The number of positive and negative results from the tested specimen was analyzed using the following formulae: Sensitivity = (number of positive samples/number of positive samples + number of false-positive samples) × 100%; specificity = (number of negative samples/number of negative samples + number of false-negative samples) × 100%.

### Results

**Figure-1:**
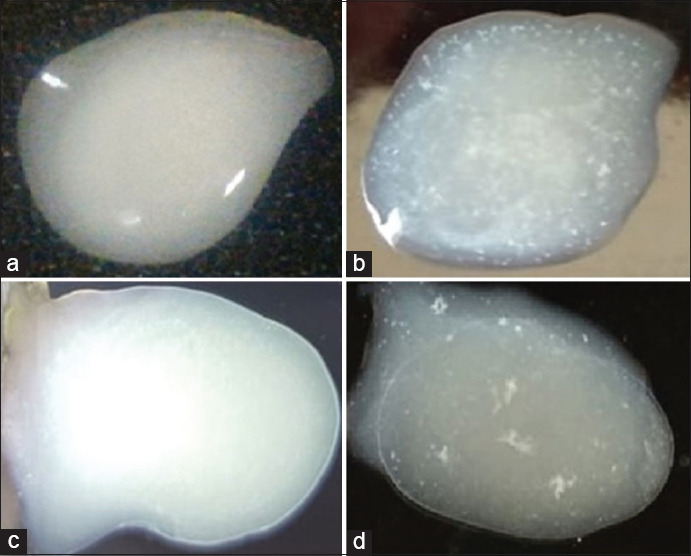
Results of coagglutination reaction against ND virus using ND coagglutination kit among the brain and fecal specimens of tested chickens from a local poultry farm in Yogyakarta. The negative result did not show the agglutination (a and c), positive sample from the brain (b) and feces (d) showed a sandy agglutination.

**Figure-2:**
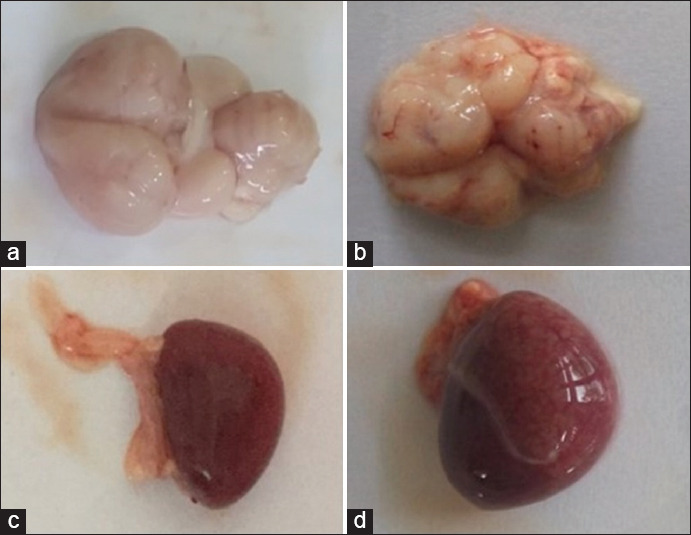
Macroscopy of the brain and spleen of tested chickens from a local poultry farm in Yogyakarta. The normal appearances of the brain (a) and spleen (c) from a negative ND infection tested with the coagglutination kit, in contrast, redness on the cerebral surface (b) and the swelling of the spleen (d) was observed from a positive ND infection tested with the coagglutination kit.

The coagglutination test showed a sandy agglutination for a positive sample (Figure-1b and d) and no agglutination for a negative sample (Figure-1a and c). Based on the coagglutination test results, 52% (13/25) of the samples showed a positive infection with the ND virus. Macroscopically, several organs, such as the brain and spleen, exhibited significant changes. The brain showed redness on the cerebral surface (Figure-2b), and the spleen was swollen (Figure-2d). These appearances are quite different from those of normal organs (Figure-2a and c).

**Figure-3:**
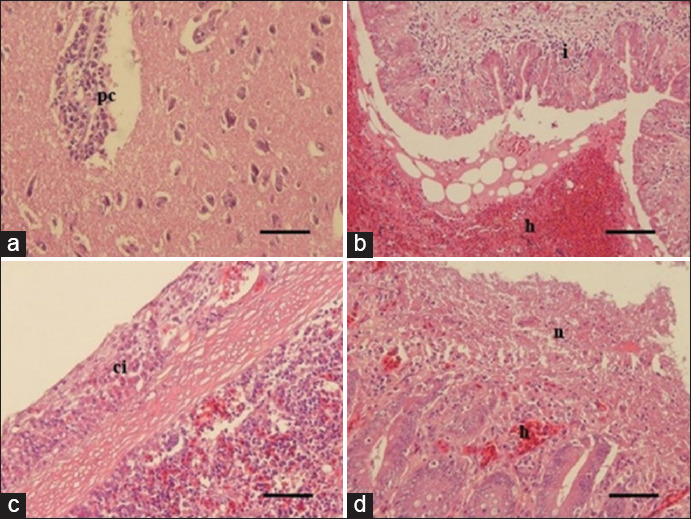
Histopathological changes of tested chickens from a local poultry farm in Yogyakarta. Perivascular cuffing (pc) surrounding the cerebral blood–brain barrier with predominant infiltration of lymphocytes that belong to ND infection (a); severe hemorrhage (h) within the lumen of parabronchus and lymphocytes infiltration (i) inside the wall of parabronchus indicated as severe hemorrhagic pneumonia (b); infiltration of lymphocytes (ci) identified in the splenic capsule (c); and hemorrhage (h) and necrosis (n) of the intestine mucosa diagnosed as necrotic hemorrhage enteritis that identified as a pathognomonic lesion of ND infection in chickens. H&E staining, 200× (a,c,d); 100× (b).

The histopathology analysis revealed that samples showing a positive result on the ND coagglutination test exhibited microscopic lesions belonging to ND infection. These samples also demonstrated several pathological changes such as perivascular cuffing surrounding the cerebral blood–brain barrier (Figure-3a), severe hemorrhagic pneumonia (Figure-3b), inflammation predominant with lymphocyte infiltration within the splenic capsule (Figure-3c, and severe necrotic hemorrhage enteritis (Figure-3d).

**Figure-4:**
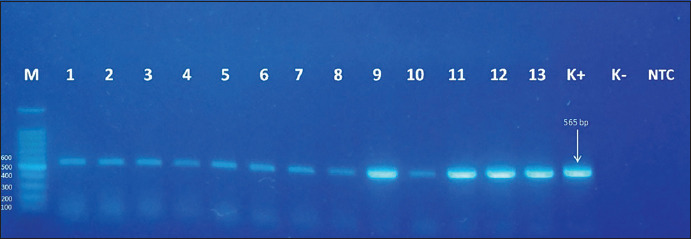
Results of RT-PCR from the positive samples that have been tested using ND coagglutination kit. M = marker, 1-3 = brain sample; 4-5 = lung sample; 6-8 = spleen sample; 9-10 = intestine sample; 11-13 = fecal sample; K+ = positive control; K− = negative control; NTC = non-template control.

**Figure-5:**
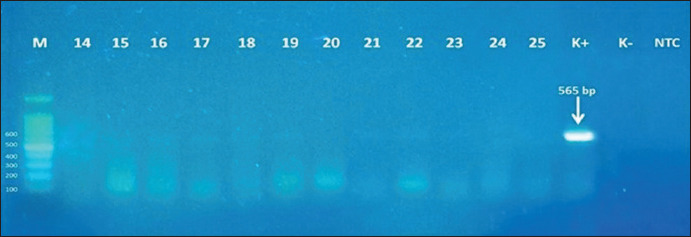
Results of RT-PCR of the negative samples that have been tested using coagglutination kit. M = marker, 14-16 = brain sample; 17-18 = lung sample; 19-20 = spleen sample; 21-22 = intestine sample; 23-25 = fecal sample; K+ = positive control; K− = negative control; NTC = non-template control.

These results were also supported by RT-PCR that indicated similar results. The RT-PCR analysis revealed a band that appeared on the 565-bp marker parallel to the positive control (Figure-4); in contrast, the negative samples did not show the appearance of the band (Figure-5). These results indicated that the coagglutination kit has high sensitivity (100%) and specificity (100%) against ND infection, which was similar to the molecular test results (Table-1). The brief details regarding the coagglutination test, molecular test, and histopathological examination of this study are presented in Table-2.

**Table 1:** The comparison of coagglutination kit and RT-PCR against ND infection from the collected specimens.

Status of the sample	RT-PCR		Total

	Positive	Negative	
ND coagglutination kit			
Positive	13	0	13
Negative	0	12	12
Total	13	12	25

RT-PCR=Reverse transcription-polymerase chain reaction, ND=Newcastle disease

**Table 2:** Comparison between the coagglutination test, molecular test, and histopathology examination of chickens in this study.

Sample code	Coagglutination kit	Molecular test	Histopathology			

			Perivascular cuffing in cerebral blood–brain barrier	Hemorrhagic pneumonia	Splenitis	Necrotic hemorrhage enteritis
1	+	+	+	+	+	+
2	+	+	+	+	+	+
3	+	+	+	+	+	+
4	+	+	+	+	+	+
5	+	+	+	+	+	+
6	+	+	+	+	+	+
7	+	+	+	+	+	+
8	+	+	+	+	+	+
9	+	+	+	+	+	+
10	+	+	+	+	+	+
11	+	+	+	+	+	+
12	+	+	+	+	+	+
13	+	+	+	+	+	+
14	−	−	−	−	−	−
15	−	−	−	−	−	−
16	−	−	−	−	−	−
17	−	−	−	−	−	−
18	−	−	−	−	−	−
19	−	−	−	−	−	−
20	−	−	−	−	−	−
21	−	−	−	−	−	−
22	−	−	−	−	−	−
23	−	−	−	−	−	−
24	−	−	−	−	−	−
25	−	−	−	−	−	−
Total positive samples	13/25 (52%)	13/25 (52%)	13/25 (52%)	15/25 (60%)	13/25 (52%)	13/25 (52%)

Positive/there is a histopathological change (+), negative (−)

## Discussion

ND is one of the prominent poultry diseases across the world. It can infect all species of poultry, including both domesticated and wild. In the poultry industry, ND has significant impacts on morbidity and mortality and decreases poultry production, which lead to severe economic losses [12,13]. The high mortality in the poultry industry due to ND infection is because this infection affects several prominent organs such as the brain, lung, digestive tract, and the lymphoid system. The brain when affected by ND shows neurological symptoms that cause uncoordinated movements [14]. In both the lung and digestive tract, ND infection causes severe bleeding along with pneumonia [15] and necrosis of the intestine that inhibits nutrient absorption and manipulates the activation of intestinal intraepithelial natural killer cells (IEL-NK) [16]. Inside the lymphoid system, the ND virus destroys lymphocytes and inhibits antibody formation, leading to decreased success of vaccination [17]. All these pathological lesions were observed in the poultry in the present study. Furthermore, the pathogenesis of ND infection can manifest in an asymptomatic manner, indicating that chickens without symptoms can act as a carrier [18]. Therefore, there is a need for rapid diagnostic tools to prevent high economic losses. A coagglutination kit can be considered as one such test [19].

The high sensitivity and specificity of the coagglutination kit compared with RT-PCR against ND infection indicated the high accuracy of the kit [20]. The agglutination reaction occurring in the coagglutination kit with the samples indicates the unique ability of protein A of *S. aureus* to bind to the Fc fragment of IgG [21]. The binding of Fc and IgG leaves the Fab fragment free to bind to antigens. Furthermore, the cell coated by the antibody reacts with the homolog antigen, which makes it possible to be visualized macroscopically [22].

For detecting ND, the secondary reaction is required to express the antigen and antibody binding. Coagglutination is based on the serological principle that involves the binding of antigen and purified immunoglobulin. During coagglutination, the immunoglobulin binds to protein A of *S. aureus* [23]. Based on its sensitivity, the coagglutination kit against ND infection can be used as a simple diagnostic tool in the poultry industry. Moreover, this coagglutination kit was compared with RT-PCR, which is a gold standard for ND detection. However, RT-PCR has few limitations, including a long detection period, requirement of high biosecurity and biosafety level, and being expensive [24]. These issues render RT-PCR inappropriate for use in endemic areas such as Indonesia.

In addition, the coagglutination test is a simple procedure and can be performed in a non-sterile area. Moreover, it is inexpensive compared with RT-PCR as a gold standard. Surprisingly, we observed that the ND coagglutination kit was reliable for testing against various samples, including organs and fecal specimens. Using fecal specimens in the diagnostic procedure are advantageous as this sample can be easily collected in a simple manner because the laboratory assistant or the veterinarian does not need to conduct invasive methods such as necropsy or tissue collection.

## Conclusion

The ND coagglutination kit is a simple, inexpensive, and rapid method exhibiting high sensitivity and specificity for the detection of ND in poultry. Moreover, this kit can be used to test fecal specimens, which avoids killing the suspected chickens. Finally, the kit can be used as a novel diagnostic tool for the detection of ND virus infection in the poultry industry.

## Authors’ Contributions

MKN, KK, TU, and YAP designed the study and drafted the manuscript. MKN, KK, TU, and YAP performed all the experimental procedures. YAP and KK conducted data analysis and interpretation. All authors read and approved on the final version of the manuscript.

## Competing Interests

The authors declare that they have no competing interests.

## Publisher’s Note

Veterinary World remains neutral with regard to jurisdictional claims in published institutional affiliation.
